# Epithelial-Stromal Polyp With Features of Perineurioma Lacking Covering Serrated Crypts Associated With a Sessile Serrated Lesion With High-Grade Dysplasia of the Colon: A Case Report

**DOI:** 10.7759/cureus.80681

**Published:** 2025-03-16

**Authors:** Hideki Mori, Kenichirou Suzuki, Masako Kawamura, Takashi Yao

**Affiliations:** 1 Pathology, Ogaki Tokushukai Hospital, Ogaki, JPN; 2 Gastroenterology, Ogaki Tokushukai Hospital, Ogaki, JPN; 3 Family Medicine, Health Check-Up Center, Ogaki Tokushukai Hospital, Ogaki, JPN; 4 Pathology, Juntendo University School of Medicine, Tokyo, JPN

**Keywords:** epithelial membrane antigen, epithelial-stromal polyp, perineurioma, proximal colon, sessile serrated lesion

## Abstract

A 79-year-old woman received a colonoscopy, and 6 polyps were found in the proximal colon. Histologically, four polyps were conventional tubular adenomas. Interestingly, one protuberant polyp was a sessile serrated lesion (SSL) with high-grade dysplasia being regarded as a potent precursor lesion for colorectal cancers. The polyp was connected with a flat-type SSL. Furthermore, another polyp was a bland spindle cell lesion filling the lamina propria. The polyp lacked covering serrated epithelium. Although the mesenchymal neoplasm displayed architectural features of perineurioma, immunoexpression of epithelial membrane antigen (EMA) and GLUT1 was negative. Accordingly, the benign mesenchymal neoplasm was considered an unusual epithelial-stromal polyp with a perineurioma-like lesion. In cases of usual perineuriomas, stromal cell proliferation is suggested to be a concern for serrated crypts that often harbor BRAF mutation. However, the mechanical nature of the proliferation of stromal cells in the polyps without serrated crypts is unknown. For the present case, proliferation of the mesenchymal cells of the epithelial-stromal polyp was suspected to relate to the serrated lesion near the polyp. Moreover, it is also presumed that the occurrence of the six polyps, including the serrated lesion, mesenchymal neoplasm, and tubular adenomas in the proximal colon, took part in both the BRAF and WNT signal pathways.

## Introduction

Gastrointestinal polyps are common lesions in daily surgical pathology practice. Some colonic polyps are known to be composed of bland spindle cells that expand the mucosa between crypts and display circumferential accentuation around polyps. These epithelial-stromal polyps have been termed benign fibroblastic polyps by some authors, whereas other authors consider them to represent perineuriomas based upon frequent immunoexpression of epithelial membrane antigen (EMA), GLUT1, and/or claudin-1 [1,2]. Most of these polyps contain an epithelial component that features serrated crypts, which often harbor BRAF mutations [2-6]. However, occasional polyps hold normal-appearing or slightly dilated crypts. Under such circumstances, colonic perineuriomas are divided into with and without crypt serration, although perineuriomas without serrated epithelium are less common [5,6]. Fundamentally, colonic perineuriomas are rare diseases, only 0.1〜1.46% of all colonic polyps are estimated as this lesion [6]. Presently, we encountered an epithelial-stromal polyp with architectural features of perineurioma. Importantly, immunoexpression of EMA was negative for this case.

Sessile serrated polyps represent the most clinically significant serrated lesion; however, the morphologic heterogeneity of dysplasia in sessile serrated polyps has only recently been identified [7]. Currently, sessile serrated polyps with dysplasia are reported to represent 2-5% of all sessile serrated polyps and <0.5% of all colorectal polyps [8,9]. Furthermore, it is now recognized that up to at least 20% of colorectal carcinomas arise not through conventional adenomas but rather through serrated polyps [7,10,11]. Serrated adenoma/polyps have been classified into sessile serrated adenomas or polyps (SSA/Ps), traditional serrated adenomas (TSAs), and hyperplastic polyps (HPs) [11]. According to the latest classification of tumors of the digestive system (WHO, 5th edition), the term sessile serrated adenoma/polyp was replaced by sessile serrated lesion (SSL) [12]. The classification also noted that dysplasia arising in an SSL most likely is an advanced polyp, regardless of the morphological grade of the dysplasia. Nevertheless, the detection of SSLs with or without dysplasia is critical, and identification by endoscopists and pathologists is inconsistent [13].

Herein, we report a rare case of a colonic epithelial-stromal polyp lacking covering serrated epithelium and EMA expression. The mesenchymal neoplasm was associated with SSL with high-grade dysplasia and tubular adenomas. The significance of the coexistence of these polyps consisting of epithelial or mesenchymal cells in a short segment of the proximal colon is discussed. In this report, detailed clinical features and differential diagnosis are not shown.

## Case presentation

A 79-year-old woman was referred to the department of gastroenterology of our hospital for receiving an endoscopic inspection since higher levels of tumor markers (CA19-9, 56.2 (standard value 0~5 ng/ml) and CEA, 5.2 (standard value 0~37U/ml)) had been noticed. During the examination, 6 polyps were found at the distance between 50 and 70 cm from the anus. Of the polyps, two lesions (No. 1 (10 mm) and No. 2 (6 mm)) were located in the ascending colon and two polyps (No. 3 (7 mm) and No. 4 (3 mm)) were present in the liver flexure. The remaining two polyps (No. 5 (3 mm) and No. 6 (4 mm)) were found in the transverse colon. The distance of the No. 1 and No. 3 polyps was 4 cm. Polyp Nos. 1, 2, and 6 were removed by a hot snare polypectomy (HSP). Nos. 4 and 5 polyps were excised by a cold forceps polypectomy (CFP). For resection of the No. 3 polyp, an endoscopic mucosal polypectomy (EMP) was used.

**Figure 1:**
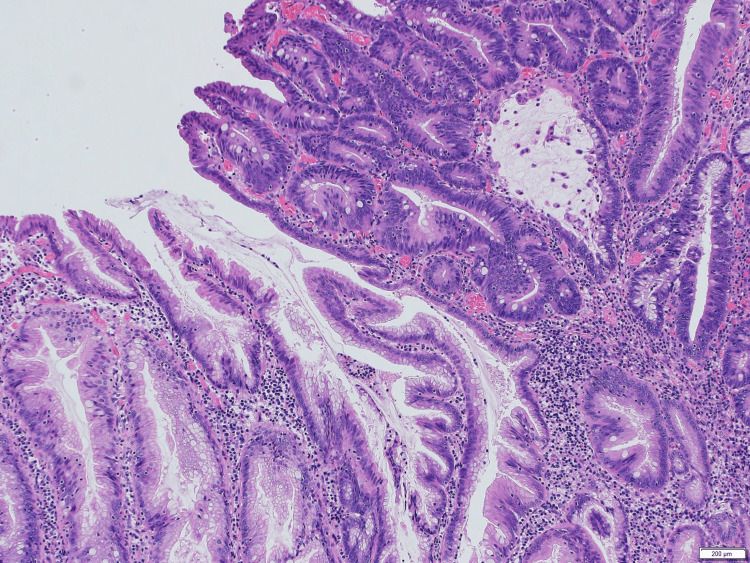
A part of SSL with high-grade dysplasia A dysplastic area (right) with an atypical cellular population is present. H.E. stain, 5x10 SSL: sessile serrated lesion; H.E.: hematoxylin and eosin

Endoscopically, all polyps except No. 1 were simple types. Polyp No. 1 was a protuberant type and displayed characteristics of SSL with high-grade dysplasia (Figure 1). In the area of dysplasia, neoplastic tubules exhibited enlarged, rounded, markedly atypical nuclei with stratification and loss of polarization.

**Figure 2:**
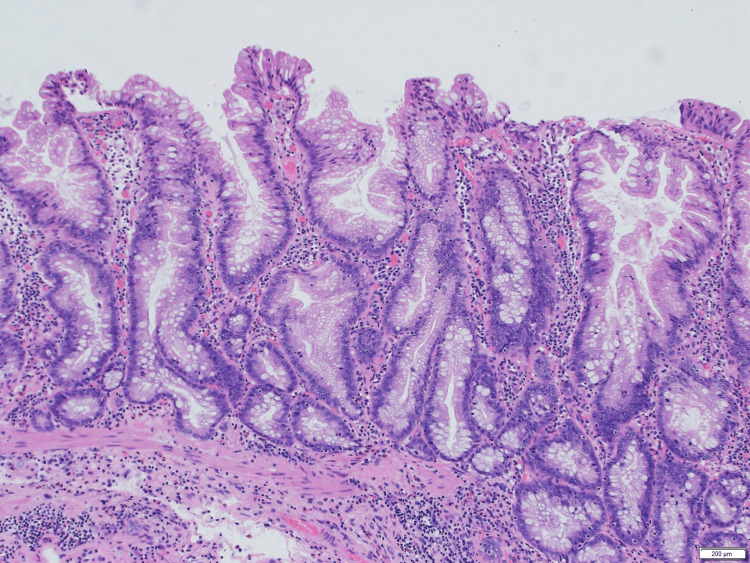
Flat-type SSL with serrated architecture H.E. stain, 5x10 SSL: sessile serrated lesion; H.E.: hematoxylin and eosin

The polyp was partly connected with a flat-type SSL (Figure 2).

**Figure 3:**
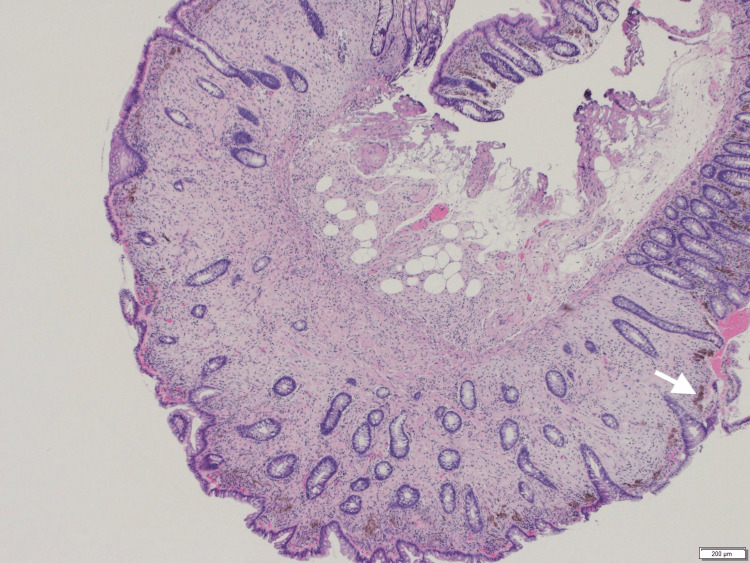
A low-power image of the epithelial-stromal polyp with features of perineurioma A proliferation of spindle cells separating non-serrated crypts is seen in the lamina propria. A hemosiderin deposit is present below the surface epithelium (arrow). H.E. stain, 5x4 H.E.: hematoxylin and eosin

Histology of No. 2, 4〜6 polyps were all tubular adenomas (low grade). Polyp No. 3 showed features of an epithelial-stromal polyp with architectural characteristics of intramucosal perineurioma. The polyp contained a poorly circumscribed spindle cell proliferation that compressed and distorted tubular crypts. No serrated crypt epithelium was confirmed (Figure 3). 

**Figure 4:**
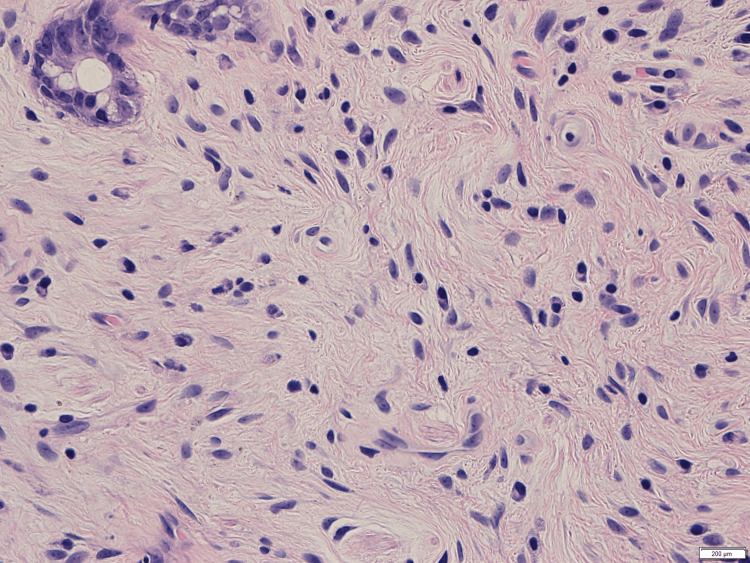
A high-power view of the epithelial-stromal polyp Spindle cells with ovoid or elongated nuclei and pale indistinct cytoplasm within a fine collagenous stroma are seen. H.E. stain, 5x20 H.E.: hematoxylin and eosin

 The spindle cells had ovoid to elongated nuclei and pale eosinophilic cytoplasm within a fine collagenous stroma (Figure 4).

**Figure 5:**
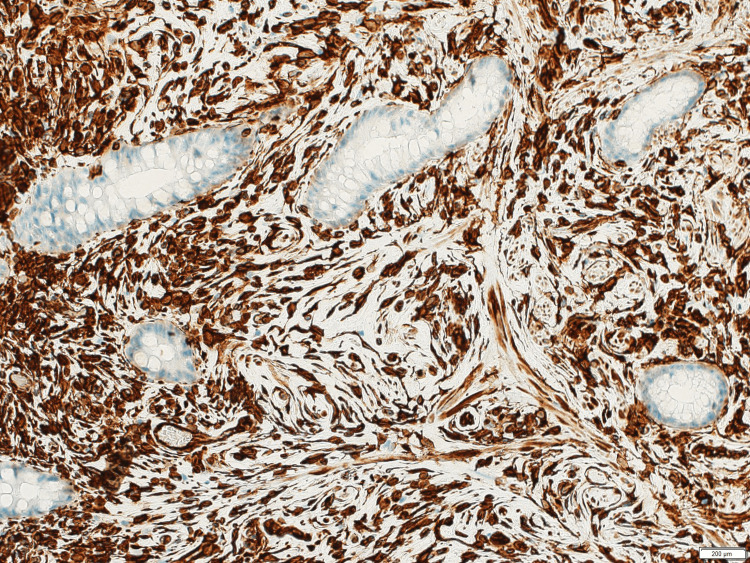
Strong immunohistochemical response of vimentin in the proliferated stromal cells of the epithelial-stromal polyp Immunostaining, 5x20

No cytological atypia or pleomorphism was present in the tumor. Hemosiderin deposition occurred only in the superficial lamina propria just below the surface epithelium. Immunohistochemically, spindle cells of the mesenchymal neoplasm revealed strong and diffuse positivity for vimentin (Figure 5) and weak reactivity for CD34. However, EMA, GLUT1, and claudin were negative. Furthermore, the absence of inflammatory cells and the negative response of c-kit, S-100, and α-SMA denied the possibility of inflammatory fibroid polyp (IFP), GIST (gastrointestinal stroma tumors), neurofibroma, schwannoma, and leiomyoma.

The low labeling index (<1%) of Ki67 suggested the benign property of this neoplasm. In the present study, an examination of the BRAF mutation was not performed since the responding antibody was not commercially available.

## Discussion

The development of sporadic colorectal cancer is thought to occur through precursor lesions that originate from either the conventional or the serrated pathway. However, an understanding of the biology and pathogenesis of serrated colorectal cancers is still poor [7,13]. Most SSLs with dysplasia develop in both the right and left colon like SSL without dysplasia and are regarded as an important precursor lesion for colorectal cancers. However, SSL with dysplasia as recognized in the present case has been observed in quite low frequency. It is reported that SSLs show an indolent growth before becoming dysplastic (>10-15 years) [14], SSLs with dysplasia presumably progress to colorectal carcinoma within a short duration [14,15]. Bettington et al. described that SSL with dysplasia occurs in patients 17 years older than in SSL without dysplasia, suggesting a long dwell time with little change in size before rapid progression to malignancy [8].

Colonic perineuriomas or fibroblastic polyps, are rare, benign mucosal lesions comprising a proliferation of bland spindle cells expressing markers of perineurial differentiation [3,4]. They preferably occur in the sigmoid colon [16,17]. The results of an immunohistochemical analysis of the present case are similar to that by Eslami-Varzaneth et al. who first described the clinicopathologic features of colorectal polyp displaying a spindle cell proliferation with only positive immunoexpression of vimentin [16]. Within a short period of time, Hornick and Fletcher reported similar cases, which they classified as perineuriomas due to immunohistochemical similarities with perineurial cells [17]. Epithelial-stromal polyps of the colon are likely divided into three types (EMA +, serrated epithelium + / EMA-, serrated epithelium-) [3]. Consequently, the present case probably belongs to that with EMA- and serrated epithelium -. The reason for the strong expression of vimentin is unknown. The expression may be specific to the proliferated mesenchymal cells of some type of epithelial-stromal neoplasm.

The pathogenesis of the epithelial-stromal neoplasm is unclear. Horneck and Fletcher reported that intramucosal perineuriomas were frequently associated with hyperplasic polyp-like changes in the adjacent or entrapped colonic epithelium, and one of the polyps was composed predominantly of a typical hyperplastic polyp within which a small focus of intramucosal perineurioma was confirmed. This evidence suggests a possibility that epithelial cell proliferation relates to the occurrence of mesenchymal neoplasia. It is also known that epithelial cells can convert into mesenchymal cells by a process known as epithelial-mesenchymal transition (EMT) [18]. Unlike the present case, perineuriomas accompany a covering serrated crypt epithelium. BRAF mutations have been demonstrated only in the serrated crypts of these epithelial-stromal neoplasms [3,4]. Although BRAF mutation analysis was not done in this case, the BRAF mutation would not appear in the covering crypts of this epithelial-stromal polyp.

Recently, Hissing and Yanttis reported that an epithelial-stromal neoplasm without a covering serrated epithelium is correlated with a negative expression of EMA and BRAF V600E. Therefore, it seems to be true that stromal cell proliferation in perineuriomas or fibroblastic polyps with a covering serrated epithelium relates to some factors elaborated by the serrated epithelium [4]. However, the mechanical nature of proliferation of stromal cells in the polyps without serrated crypts is unknown. In this case, an epithelial-stromal polyp lacking serrated crypts was located only 4 cm toward the distal direction from the serrated lesion with high-grade dysplasia. Therefore, the authors of this case report assume that some factors generated by a neighboring serrated lesion caused the epithelial-mesenchymal interactions leading to spindle cell proliferation in the polyp without a covering serrated epithelium. Such an assumption regarding the proliferation of mesenchymal cells in the epithelial-stromal neoplasm has not been described.

It is reported that overexpressed MicroRNA-147 inhibited EMT and downregulated the expression of β-catenin and c-myc, which were related to the WNT/β-catenin pathway [19]. Pai et al. described that WNT signaling pathway activation occurs in all pathways through different mechanisms, at the transition to dysplasia or earlier in traditional serrated adenoma [7]. Furthermore, it is confirmed that WNT/β-catenin signaling activation is concerned with the serrated neoplasia pathway as well as the adenoma-carcinoma sequence in the colorectum [20]. Accordingly, it is probable that the induction of the epithelial-stromal neoplasm as well as the epithelial neoplasms in the colon in this case were related to both BRAF-related and WNT signal pathways.

## Conclusions

An epithelial-stromal polyp with features of perineurioma lacking covering serrated crypts and a sessile serrated lesion with high-grade dysplasia were recognized in the proximal colon. Two lesions were located next to each other. It is presumed that histogenesis of the epithelial-stromal polyp was related to the presence of the serrated lesion.

## References

[1] Groisman GM, Polak-Charcon S (2008). Fibroblastic polyp of the colon and colonic perineurioma: 2 names for a single entity?. Am J Surg Pathol.

[2] Agaimy A, Stoehr R, Vieth M, Hartmann A (2010). Benign serrated colorectal fibroblastic polyps/intramucosal perineuriomas are true mixed epithelial-stromal polyps (hybrid hyperplastic polyp/mucosal perineurioma) with frequent BRAF mutations. Am J Surg Pathol.

[3] Hissong E, Yantiss RK (2021). Epithelial-stromal polyps of the colon are not perineuriomas. Am J Clin Pathol.

[4] Pai RK, Mojtahed A, Rouse RV (2011). Histologic and molecular analyses of colonic perineurial-like proliferations in serrated polyps: perineurial-like stromal proliferations are seen in sessile serrated adenomas. Am J Surg Pathol.

[5] Groisman GM, Hershkovitz D, Vieth M, Sabo E (2013). Colonic perineuriomas with and without crypt serration. A comparative study. Am J Surg Pathol.

[6] Jama GM, Evans M, Fazal MW, Singh-Ranger D (2018). Perineurioma of the sigmoid colon. BMJ Case Rep.

[7] Pai RK, Bettington M, Srivastava A, Rosty C (2019). An update on the morphology and molecular pathology of serrated colorectal polyps and associated carcinomas. Mod Pathol.

[8] Bettington M, Walker N, Rosty C (2017). Clinicopathological and molecular features of sessile serrated adenomas with dysplasia or carcinoma. Gut.

[9] Liu C, Walker NI, Leggett BA, Whitehall VL, Bettington ML, Rosty C (2017). Sessile serrated adenomas with dysplasia: morphological patterns and correlations with MLH1 immunohistochemistry. Mod Pathol.

[10] The Cancer Genome Atlas Network (2012). Comprehensive molecular characterization of human colon and rectal cancer. Nature.

[11] Phipps AI, Limburg PJ, Baron JA (2015). Association between molecular subtypes of colorectal cancer and patient survival. Gastroenterology.

[12] Nakanishi Y, Diaz-Meco MT, Moscat J (2019). Serrated colorectal cancer; the road less travelled?. Trends Cancer.

[13] Pai RK, Makinen MJ, Rosty C (2019). Colorectal serrated lesions and polyps. Digestive System Tumours. WHO Classification of Tumours, 5th Edition, Volume 1.

[14] Murakami T, Kurosawa T, Fukushima H, Shibuya T, Yao T, Nagahara A (2022). Sessile serrated lesions: clinicopathological characteristics, endoscopic diagnosis, and management. Dig Endosc.

[15] Uthumi T, Yamada Y, Diaz-Meco T, Moscat J, Nakanishi Y. (2023). Sessile serrated lesions with dysplasia; is it possible to nip them in the bud?. J Gastroenterol.

[16] Eslami-Vazaneh F, Washington K, Robert M, Kashgarian M, Goldblum J, Jain D (2004). Benign fibroblastic polyps of the colon. A histologic, immunohistochemical and ultrastructural study. Am J Surg Pathol.

[17] Hornick JL, Fletcher CDM (2005). Intestinal perineuriomas. Clinicopathologic definition of a new anatomic subset in a series of 10 cases. Am J Pathol.

[18] Thiery JP and Sleeman J (2006). Complex networks orchestrate epithelial-mesenchymal transitions. Nature Rev Mol Cell Biol.

[19] Ning X, Wang C, Zhang M, Wang K (2019). Ectopic expression of miR-147 inhibits stem cell marker and epithelial-mesenchymal transition (EMT)-related protein expression in colon cancer cells. Oncol Res.

[20] Murakami T, Mitomi H, Saito T (2015). Distinct WNT/β-catenin signaling activation in the serrated neoplasia pathway and the adenoma-carcinoma sequence of the colorectum. Mod Pathol.

